# The Influence of Diabetic Peripheral Neuropathy on the Duration of Sciatic Nerve Block with 1.3% Liposomal Bupivacaine and 0.25% Bupivacaine Hydrochloride in a Mouse Model

**DOI:** 10.3390/pharmaceutics14091824

**Published:** 2022-08-30

**Authors:** Liljana Markova, Erika Cvetko, Chiedozie Kenneth Ugwoke, Simon Horvat, Nejc Umek, Tatjana Stopar Pintarič

**Affiliations:** 1 Department of Anaesthesiology and Surgical Intensive Therapy, University Medical Centre Ljubljana, Zaloška Cesta 7, 1000 Ljubljana, Slovenia; 2 Institute of Anatomy, Faculty of Medicine, University of Ljubljana, Korytkova Ulica 2, 1000 Ljubljana, Slovenia; 3 Department of Animal Science, Biotechnology and Immunology, Biotechnical Faculty, University of Ljubljana, Groblje 3, 1230 Domžale, Slovenia

**Keywords:** peripheral nerve block, local anesthetics, liposomal bupivacaine, peripheral neuropathy, diabetes

## Abstract

Little is known regarding the pharmacological properties of extended-release local anesthetics in the setting of diabetic peripheral neuropathy. We investigated and compared the duration of sciatic nerve block following administration of clinically relevant concentrations of liposomal bupivacaine (LB) and bupivacaine hydrochloride (BH) in diabetic mice with peripheral neuropathy. In this prospective, randomized, and double-blind study, twenty-four female C57BL/6J-OlaHsd mice were assigned to a streptozotocin-induced type 1 diabetes group and a control group without diabetes. The presence of peripheral neuropathy was established by assessing the duration of thermal latency of the plantar and tail-flick tests, following which both groups were subdivided into two subgroups in which 35 mg/kg of 1.31% LB and 7 mg/kg of 0.25% BH were respectively administered for sciatic nerve block. The average sensory block duration with BH was 106 min and 117.1 min in the control and diabetic groups, respectively. With LB, the average sensory block duration was 118 min in the control mice, while in mice with diabetic peripheral neuropathy, the average block duration was significantly longer and above the 270 min limit set in our study. Accordingly, sensory block duration was longer with LB compared to BH, and diabetic peripheral neuropathy significantly increased sciatic nerve block duration with LB.

## 1. Introduction

Diabetes mellitus (DM) describes a group of heterogeneous diseases typically characterized by hyperglycemia. Clinically, it is defined as a fasting venous plasma glucose level of ≥126 mg/dL (7.0 mmol/L), a random plasma glucose level of ≥200 mg/dL (11.1 mmol/L) in a patient with classical symptoms of hyperglycemia, a venous plasma glucose level of ≥200 mg/dL (11.1 mmol/L) 2 h after a 75-g oral glucose tolerance test, or a glycated hemoglobin level of ≥6.5% (≥48 mmol/mol Hb). DM may be classified into four general categories. DM type 1 describes an abnormality in insulin secretion due to predominantly immunologically-driven pancreatic β-cell destruction often resulting in absolute insulin deficiency. DM type 2, the most prevalent form of the disease, describes a progressive impairment of insulin secretion on the background of insulin resistance. The other types of DM include gestational DM (glucose tolerance disorder first diagnosed in the second or third trimester of pregnancy), and other specific forms of DM, such as monogenic DM syndrome, or DM secondary to diseases of the exocrine pancreas, endocrine organs, drugs, chemicals, or infections [1,2]. Several pathophysiological mechanisms are implicated in the development of DM and its complications, including mitochondrial dysfunction, oxidative stress, inflammation, accumulation of ectopic lipid metabolites, immune dysregulation, vascular dysfunction, and gene mutations [3,4,5].

Over the past 20 years, the number of people with DM worldwide has doubled, with global prevalence projected to escalate further over the next few decades [6,7,8]. The chronic nature of the disease and the involvement of all organ systems are the primary reasons behind the associated high morbidity and mortality and the enormous economic burden [9,10]. Diabetic peripheral neuropathy is one of the typical complications of DM, affecting approximately 50% of all diabetic patients and underlying the pathogenesis of several clinical complications, including diabetic foot syndrome [11]. Surgical treatment is frequently indicated in acute and chronic complications related to DM, and regional anesthetic techniques are often favored for surgical anesthesia and pain control in diabetic patients [12]. For example, sciatic nerve blocks are commonly performed for procedures such as below-knee amputations and foot and ankle surgery, which are common in diabetic patients [13,14]. Special pre-and peri-operative anesthetic considerations are often required for these patients, and the choice of appropriate anesthetic modality is typically a complex process requiring careful calibration of the patient’s risk-benefit profile to mitigate peri- and post-operative complications.

To enhance the efficacy of peripheral nerve blocks, methods that extend analgesia while decreasing anesthetic dosage and adverse effects are required. In recent years, the addition of different adjuvants (e.g., epinephrine, opioids, alpha-2 agonists) for prolongation of a sensory block was introduced and widely implemented in both catheter and single-shot techniques [15,16,17]. However, data on the prolongation of peripheral nerve block by adjuvants are conflicting, and reliable extension of sensory block duration beyond 24 h remains elusive with conventional agents [18]. It is known that different types of adjuvants are associated with toxicity-related clinical complications [17]. Hypotension, bradycardia, and sedation are important side effects of alpha-2 agonists, while pruritus, nausea, and somnolence are associated with opioid adjuvants [16]. In diabetic patients with peripheral neuropathy, epinephrine is associated with both systemic side effects, such as hypertension and tachycardia, as well as local effects on disease-modified nerves and tissues [15]. To reduce the above-mentioned side effects and to achieve a prolonged duration of peripheral nerve blocks, extended-release local anesthetic formulations have been developed. The five major categories of these local anesthetic vehicle formulations are injectable particles, injectable liquids, hybrid systems, macroscopic devices, and triggerable formulations. Prolongation of local anesthetic effect has been achieved by a wide range of delivery systems, but with a broad variation in terms of duration and intensity of regional anesthesia [19]. Liposomes are widely utilized injectable, nano-fabricated drug delivery systems [20], with many advantages when used as a local anesthetic carrier. Liposomes as a carrier proved neutral or benign regarding tissue reaction despite the associated improvement of local anesthetic properties such as enhanced duration of action, reduced circulating plasma levels, and reduced central nervous system and cardiovascular toxicity [19]. Different liposome-encapsulated local anesthetic formulations have been evaluated for various purposes [21,22]. However, only a few have managed to enter clinical trials, among which the oldest and the most widely investigated is bupivacaine liposome injectable suspension (EXPAREL^®^) [19,23].

Since its initial approval in 2011 for surgical site infiltration, the clinical application of EXPAREL^®^ has expanded [24]. In 2018, it was approved for interscalene brachial plexus block; in 2020, for field block infiltration, brachial plexus block, and femoral nerve block; and in 2021, for local infiltration in pediatric patients aged six years and above. A few studies have investigated liposomal bupivacaine (LB) using different animal models [25,26,27,28]. Among the first published reports examining encapsulated bupivacaine are studies from Grant et al. [29,30], which evaluated the duration of analgesia following the application of LB in a mouse model. McAlvin et al. [27] compared the time of sensory block between 0.5% bupivacaine hydrochloride (BH) and 1.3% LB. The results of their study, as well as the analysis of Markova et al. [31], presented LB as the preferred local anesthetic regarding neurotoxicity potential. However, the studies mentioned above examined the duration of action of LB in non-damaged nerves. The effects of local anesthetics on nerves altered by DM have remained a subject of debate. While a prolonged duration of action of local anesthetics in peripheral nerves damaged by DM has been reported [32,33,34], limited knowledge exists regarding the duration of sensory block induced using LB. Both preclinical and clinical studies are currently scarce on the use of extended-release local anesthetics in the context of diabetic peripheral neuropathy. Accordingly, in the present study, we applied analytical methods to investigate and compare the duration and intensity of sciatic nerve block with LB and BH in the setting of peripheral neuropathy due to DM type 1, to provide data to guide the rational use of local anesthetic modalities in pathologic nerve conditions.

## 2. Materials and Methods

The study was designed as a prospective, randomized, and double-blind experiment on a mouse model of peripheral neuropathy due to DM type 1 and conducted in strict compliance with the recommendations of the National Institutes of Health’s Guide for the Care and the Use of Laboratory Animals [35]. All research protocols were reviewed and approved by the Ethical Committee for Laboratory Animals of the Republic of Slovenia (Permit Number: U34401-21/2013/6) following the European Union directives on the use of laboratory animals in research and the ARRIVE guidelines [36].

### 2.1. Experimental Animals

The study included 24 six-week-old female C57BL/6J-OlaHsd mice *(Mus musculus)* with a body mass range of 25–30 g, obtained from Harlan Laboratories—Envigo (Italy) and reared at the Centre for Laboratory Animals of the Biotechnical Faculty of the University of Ljubljana. The selected female mouse model develops DM faster and with greater severity than males and expresses most of the typical neuropathic characteristics of DM type 1 in humans [37]. The animals were housed in individually ventilated cages maintained at a temperature of 23 ± 1 °C, 40–60% humidity, and a 12-h light-dark cycle. After two weeks of acclimatization with free access to water and a standard diet (Mucedola, Milan, Italy), all mice were weighed to the nearest 1 g and then randomly divided into two groups: the streptozocin (STZ)-induced DM group and a control group without DM. STZ induction of DM type 1 was performed per standard protocols [37,38]. Four hours prior to STZ injection, the animals had no access to food but had free access to water. Immediately before administration, STZ was dissolved in sodium citrate buffer (pH 4.5) to a 10 mg/mL final concentration. Mice from the experimental group were injected intraperitoneally with 200 mg/kg of dissolved STZ; mice from the control group got the same volume of citrate buffer (pH 4.5). STZ is an alkaline substance that destroys beta cells of the pancreas and is considered the most appropriate and reproducible modality for DM induction in animals [39]. After the STZ application, the animals were returned to their cages with free access to food and water with 10% sucrose. According to protocol [37], 10% of sucrose prevents fatal hypoglycemia, which can occur with massive beta-cell necrosis and the release of large amounts of insulin. On the third day after intraperitoneal administration, water with sucrose was replaced with plain water. Mice were observed daily for signs of pain or distress, and changes in breathing, appetite, and activity (e.g., lethargy or hyperactivity). We measured the consumed food and water daily and replaced them accordingly. Upon dehydration, weight loss over 10% of baseline, and glucose concentrations > 30 mmol·L^−1^ (fasting glucose levels were measured with a Bayer Contour glucometer (Ascensia Diabetes Care Holdings AG, Basel, Switzerland), mice were subcutaneously hydrated with 1 mL of saline and treated with insulin (0.1 to 1.2 U kg^−1^ daily, depending on glucose concentration). At four weeks after STZ treatment, mice with fasting blood glucose levels > 23 mmol·L^−1^ were included in the study as diabetic; in the case of glucose levels < 8 mmol·L^−1^, mice were considered non-diabetic and excluded from the study [38].

### 2.2. Assessment of Peripheral Neuropathy

The Animal Models of Diabetic Complications Consortium (AMDCC) Neuropathy Phenotyping Protocols suggest that the threshold of heating and cooling perception is a sensitive indicator of early polyneuropathy [40,41,42]. Accordingly, following confirmation of diabetic status at four weeks post-STZ treatment [43,44], we tested the duration of thermal latency, defined as the time of paw withdrawal from thermal stimuli. For this purpose, we used the plantar test (IITC Plantar Analgesia Meter, Woodland Hills, CA, USA) with an infrared heat intensity of 50% and the ‘tail flick’ test with an intensity of 40%. To prevent thermal tissue damage and secondary hyperalgesia, we determined the time of heat stimulus withdrawal (‘cut-off’) for the plantar test of 15 s and the ‘tail flick’ of 4 s [45]. The plantar test was performed twice: before STZ application and two days before the sciatic nerve block, while the ‘tail flick’ test was only before the sciatic block procedure. The heat stimulus was repeated three times at 5-min intervals [46]. The mean value was calculated by considering values within one standard deviation from the average of all measurements.

### 2.3. Experimental Groups

Twenty-four female mice (12 with DM type 1 and confirmed peripheral neuropathy and 12 controls without DM and peripheral neuropathy) were included for the sciatic nerve block procedure. Both groups were then divided into two subgroups in which 35 mg kg^−1^ 1.31% LB (EXPAREL^®^, Pacira Pharmaceuticals Inc., Parsippany, NJ, USA) and 7 mg kg^−1^ 0.25% BH (Marcain Spinal^®^, AstraZeneca UK, Ltd., Cambridge, UK) (according to the Institutional Animal Care and Use Committee, University of California, San Francisco, CA, USA) were used [26,27].

### 2.4. Performance of Sciatic Nerve Block

Inhalational anesthesia with isoflurane in a mixture of oxygen and nitric oxide through a face mask was administered before the sciatic nerve block. The body temperature of the mice was maintained at 37 °C using a hot plate. The sciatic nerve block was performed percutaneously by injection of local anesthetics using a 29-GA needle (Omnican^®^, B. Braun Melsungen AG, Melsungen, Germany). During the injection of local anesthetics, the mouse was put in a lateral position so that the leg on which the block was performed formed a right angle with the longitudinal axis of the torso. The needle was introduced posteromedially in the direction of the greater trochanter of the femur and directed anteromedially. The local anesthetics were injected upon contact of the needle with the bone [27]. Eighty-five microliters of local anesthetics was used to achieve a sufficient dose of LB and BH.

### 2.5. Determination of Nerve Block Duration

The duration of sciatic nerve blocks of the mice within each treatment (LB or BH) and condition (peripheral neuropathy due to DM or control) were distributed using a balanced incomplete block design (BIBD). The design considered four treatments and 180 measuring points. Each mouse was measured once within 13.5 min, while the remaining mice from the same treatment and condition were distributed at different time points within the same interval. The total analyzed duration for each treatment and condition was 270 min. The total block duration was determined based on the study by McAlvin et al. [27], where the authors measured the nerve block in rodents anesthetized with LB for a total interval of 240 min. Within each treatment and condition, measurements were recorded every 1.5 min in different subjects per condition and treatment from the beginning of the administration of local anesthetics. If the animal had not removed the paw from the light beam within 15 s, the heat stimulus was removed to avoid damage and the development of secondary hyperalgesia. The measurement of thermal latency and the sensory block duration was made using the Plantar Test Analgesia Meter.

### 2.6. Data Analysis

The descriptive data are presented as mean and standard deviation (SD) for the continuous variables or as numbers and percentages for categorized variables. The duration of the sciatic nerve block was calculated by the method used by Grant and colleagues [47], Davis et al. [48], and Yu et al. [49].

For each mouse at each time interval, the percentage of maximal possible effect (% MPE) of local anesthetic was calculated.

$$\%\;\mathrm{MPE}=\frac{\mathrm{Postinjection}\;\mathrm{latency}\;-\;\mathrm{Preinjection}\;\mathrm{latency}}{\;\mathrm{Cutoff}\;\mathrm{time}\;\left(15\;s\right)-\;\mathrm{Preinjection}\;\mathrm{latency}}\times 100\;\%$$

To determine the duration of the sensory block, a mean % MPE score for each treatment and condition within the interval of 13.5 min was calculated. We fit the 3rd order polynomial function to create a curve with interpolation using the data for the average group % MPE calculated in the selected time interval. The global maximum, referring to the onset of action of local anesthetics, and local and global minimum, referring to the release and total duration of local anesthetics, were used to describe the pattern of behavior of the local anesthetics in both conditions. Where the average % MPE per treatment and condition was below 15%, the sensory block was considered diminished. In the preparation of curve fitting graphs, we used the Windows version of GraphPad Prism 9.4.0. MPE recorded over time was also used to determine the receiver operating curves (ROC) per condition and treatment. Two ROC curves were constructed per condition and treatment. Two-by-two contingency tables were created to calculate true- and false-positive rates for block release to differentiate between treatments and conditions. We plotted the true-positive rate against the false-positive rate for an estimate to predict the adverse outcome to develop the ROC curves. The areas under the curves and the standard error (SE) were estimated by a point-to-point trapezoidal method of integration [50,51]. To compare the areas under the ROC curves per treatment and condition, χ^2^ statistics were used [52]. A two-sample independent t-test was used to analyze the difference between treatments and conditions in terms of general characteristics linked to the presence of the condition. All statistical analyses were performed using STATA (version 17.0; StataCorp LLC, College Station, TX, USA). The outcomes were statistically significant at *p* < 0.05 and notably significant at *p* < 0.1.

## 3. Results

All mice injected intraperitoneally with STZ had blood glucose levels of 23 mmol·L^−1^ or greater and were accordingly classified as diabetic. Prior to the induction of DM, there was no difference between the mean weight of the diabetic group (23.5 g ± 1.7) and the nondiabetic group (24.1 g ± 1.7) (Table 1). Four weeks after STZ injection, the diabetic mice lagged in weight: 21.3 g ± 2.4 for diabetic mice versus 28.5 g ± 2.3 for nondiabetic mice (*p* < 0.001). Diabetic mice also had polyuria, assessed based on the wetness of the cage bedding. The duration of thermal latency of the plantar test before sciatic nerve block was significantly increased (11.9 s ± 3.4; *p* < 0.001) in the diabetic peripheral neuropathy group compared to the control group (6.4 s ± 2.8; *p* < 0.001). A ‘tail flick’ test performed before the sensory experiment showed a notable difference (*p* < 0.1) in the duration of thermal latency between mice with peripheral neuropathy due to DM (2.2 s ± 0.4) and control mice (1.9 s ± 0.4). Three diabetic mice from the BH and one diabetic mouse from the LB group with a degree of peripheral neuropathy equal to or greater than the ‘cut-off’ time were excluded from the analysis.

**Figure 1:**
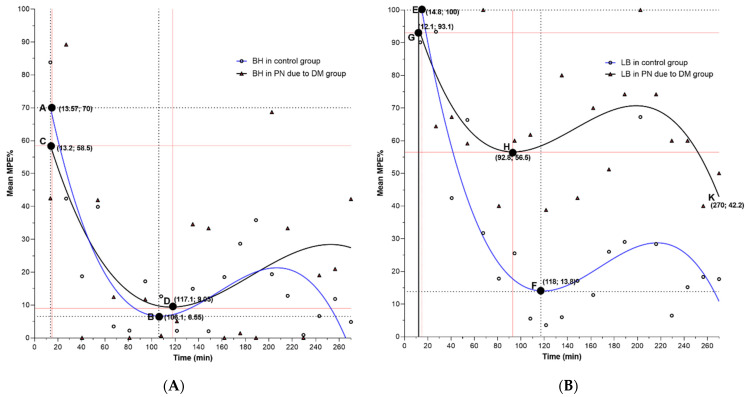
Relationship between mean % MPE and time sequence (13.5 min) for the control group and mice with peripheral neuropathy due to DM treated with bupivacaine hydrochloride (BH) (**A**) and liposomal bupivacaine (LB) (**B**): The curves are drawn with interpolation using 3rd order polynomial functional model. The initial MPE % is marked with capital letters A, C, E, G, and the block release with letters B, D, F, H, and K. The raw data, including means and variability, is presented in the Supplementary Material (Supplementary Table S1). DM—diabetes mellitus, PN—peripheral neuropathy MPE—maximal possible effect.

The results presented in Figure 1 and Supplementary Table S1 show the duration and mean (SD) % MPE of BH and LB in the control and diabetic peripheral neuropathy mice. Using the 3rd order polynomial model, we interpolated the data to fit a curve to describe the behavioral pattern of the selected local anesthetic for the control and diabetic peripheral neuropathy groups. The results show that BH in the control group had an onset time of 13.57 min with an average MPE of 70%, while in the diabetic peripheral neuropathy group, the onset time was 13.2 min with MPE of 58.5%. At 14.8 min following the injection of LB, we observed MPE of 100% in the control group, while 12.1 min was needed to observe MPE of 93.1% in the group with peripheral neuropathy due to DM. The estimated duration of the BH was 106 min in the control and 117.1 min in the diabetic peripheral neuropathy group. The control mice that received LB had a sensory block duration of 118 min, while in those with peripheral neuropathy due to DM the first release of the block was at 56.5% MPE and occurred at 92.8 min. At 200 min, we observed a sharp decline in the average % MPE, which reached the minimum MPE at 270 min, suggesting that the block lasted above the limit of 270 min set for our study. In the control group, the 3rd order polynomial model explained 70.5% of the variance of the data for BH and 72.9% for LB. The diabetic peripheral neuropathy models have coefficients of determination of 24.5% and 29.0% for BH and LB, respectively. The results presented in the figures show that although peripheral neuropathy may generally prolong sensory block duration irrespective of which local anesthetic was used, LB remarkably increases the block duration in mice with peripheral neuropathy due to DM.

**Figure 2:**
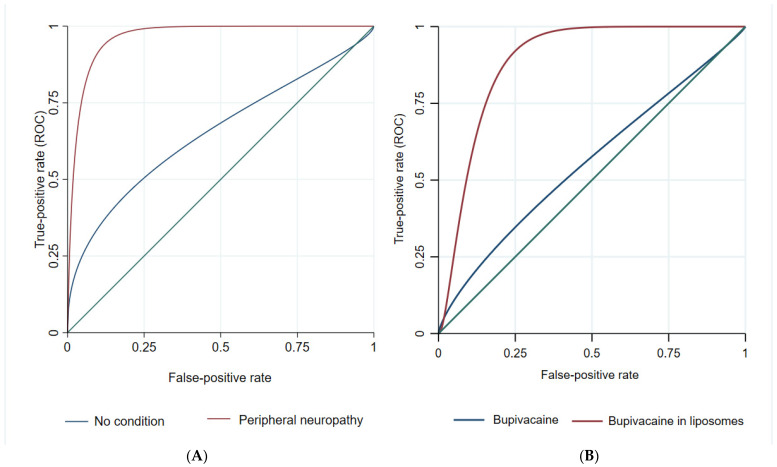
(**A**) The relationship between % MPE and probability of correctly classifying mice treatment based on the type of condition. (**B**) The relationship between % MPE and probability of correctly classifying mice condition based on the type of local anesthetics.

Based on the type of condition and type of local anesthetic, by measuring the % MPE, we developed ROC curves to correctly predict between mice with different conditions and those receiving different types of local anesthetic. The results show a higher discrimination capacity in the model describing the behavior of mice with peripheral neuropathy due to DM and mice receiving LB (Figure 2).

Table 2 presents the ROC model results. The analysis shows a significant difference between models developed upon the different types of local anesthetics and conditions in terms of correctly predicting the duration and pattern of behavior in the mice model. The LB model shows significantly higher discrimination capacity based on the different types of conditions compared to the model where BH is present. Furthermore, the model in which peripheral neuropathy due to DM is present shows better prediction capacity for the duration and the pattern of behavior irrespective of which local anesthetic is used compared to the model in which the control condition was analyzed. This analysis shows a similar pattern of behavior in terms of thermal latency between mice with peripheral neuropathy due to DM, regardless of which local anesthetic is applied and a similar pattern of behavior when LB is used, irrespective of which condition is present.

## 4. Discussion

We demonstrated that LB provided a longer sensory block than BH, irrespective of whether the nerve is damaged by DM, and that diabetic peripheral neuropathy significantly increased the duration and intensity of nerve block with LB. Several preclinical and clinical studies have compared LB and BH in terms of duration of action, systemic and local tissue toxicity, patient satisfaction and cost, and prolonged duration of LB-induced nerve block has already been confirmed in different animal models [27,28,49,53], consistent with our findings. However, the exact duration of nerve block induced by local anesthetics is difficult to determine and is generally affected by different factors, including the type of local anesthetics and type of encapsulation, method of determination, animal model, and sample size in relation to the type of disease condition, site of application and concentration. Sample size, method of application, and method of determination influence the accuracy of the estimates, while the nerve condition and the local anesthetics type, concentration, and different modalities of prolongation (e.g., adjuvants, type of local anesthetic encapsulation) influence the duration of a nerve block. In terms of sample size, our study follows the number of mice used in the studies of Kroin [33], Davis [48] and Sousa [54], which is also comparable with the studies of Grant [30,55] and de Araujo [56]. To minimize the impact of all factors that can affect the accuracy of estimates, we selected a percutaneous, closed model for performing sciatic nerve blocks. This model also avoids the possibility of additional nerve damage by the immune response that can occur during the open model [25], which could affect the duration of local anesthetics. The model has proven to be appropriate in other studies, offering minimal systemic and local immunological reaction, minimal nerve damage, and minimal possibility of infections and other complications compared to the open model [27,31].

Determination of nerve block duration was made following the method of Grant et al. [47], which is suitable for mice with evident peripheral neuropathy. Mice with a degree of neuropathy equal to or higher than the ‘cut-off’ time were excluded from the calculation. To determine the duration of the nerve block, some studies have used mean and median values calculated based on the individual estimate. The method of calculation may influence the final data even if the same concentrations of local anesthetics and animal models are used [27,47]. The duration of sciatic nerve block induced with the selected concentration for BH in control mice proved to be the same as the duration at the same concentration of BH in the study of de Araujo et al. [56]. This validates the performance and measurements of sciatic nerve block induced with local anesthetics in our study. We settled at 0.25% BH as a mean percentage of bupivacaine from a previously published study in mice that reported increasing duration of the block with an increase of concentration from 0.125 or 0.25 to 0.5% [56]. Both 0.25% BH and 1.3% LB are concentrations of these agents relevant to clinical practice. Furthermore, the concentration of 0.25% BH was selected as it is the more comparable concentration with 1.3% LB. This is due to the very low initial exposure of the nerve to free bupivacaine (only 3% is in extracapsular form) after LB application, which is comparable to the lowest concentration of 0.25% BH in clinical use. A higher concentration of local anesthetics provides a faster onset of action and longer duration of peripheral nerve block; however, tissue and nerve toxicity with higher concentrations have also been detected [57,58,59,60,61]. Concerns for neurotoxicity are even more significant in the setting of preexisting neurological disease [62]. Preclinical and clinical studies have demonstrated alterations in the time of onset, mechanism, and duration of action of local anesthetics in normal and damaged nerves [63,64]. Our results are consistent with preclinical studies in rats, where nerve block prolongation ranged from 25 to 50% in diabetic rats with sensory neuropathy compared to healthy rats [32,33,34,65].

While arguments regarding the prolongation of peripheral nerve block duration in diabetic peripheral neuropathy exist, the actual cause is unclear. DM is associated with a wide range of peripheral neuropathy syndromes, ranging from asymptomatic distal sensory neuropathy to severe neuropathy with plexus and root involvement, and it has been shown that the duration of the disease and the degree of glycemic control are key factors that affect the severity of peripheral neuropathy [66]. Microvascular damage to nerve fibers, abnormalities in calcium homeostasis, decreased activity of potassium and sodium channels of nerve fibers, loss of myelinated and unmyelinated fibers, axonal degeneration, failed axonal regeneration, and collateral budding of axons are just some of the most characteristic pathophysiological changes of diabetic peripheral neuropathy described in rats [67,68,69]. Studies of diabetic patients with peripheral neuropathy have described changes in the threshold of evoked motor responses, conduction velocity, and nerve sensitivity to local anesthetics during and after peripheral nerve block [70]. Baeriswyl et al. [63] reported a prolonged duration of nerve block in patients with DM type 2 and peripheral neuropathy compared to patients without peripheral neuropathy, while other clinical studies have also highlighted the prolongation of nerve block duration in diabetic compared to non-diabetic patients [70,71]. In this context, it is debatable whether hyperglycemia itself may be the cause of the prolonged duration of sensory nerve block. In 2010, Perkins et al. [72] demonstrated a significant improvement in sensory conduction of nerves after lowering the concentration of glycated hemoglobin in a group of patients with sensory and motor neuropathy. Based on this, we can infer that the duration of peripheral block in patients with DM and poor glycemic control will be longer compared to patients with DM and well-controlled glycemia [73]. The results of research done in this area highlight that glycemic control is directly related to the risk of developing and progressing microvascular complications (retinopathy, nephropathy, and neuropathy) in patients with DM type 1 and type 2 [74,75]. Increased nerve block effect in patients with DM and peripheral neuropathy, increased nerve sensitivity to local anesthetics, and slower local anesthetic leaching with concomitantly impaired microcirculation support the assumption of local anesthetics dose reduction, although this has not been extensively investigated or confirmed in clinical trials [32,63,64].

Previous studies on rodents showed the prolonged duration of sciatic nerve block in animals treated with LB compared to BH [27]. Results in our study are in accordance with these findings: the duration of sciatic nerve block induced with LB versus BH was longer in both the diabetic group with peripheral neuropathy and the control group, albeit more prolonged in the former. Considering that our study is the first to investigate and analyze peripheral nerve block induced by LB in mice with diabetic peripheral neuropathy, we can comment with caution that LB induced denser and more consistent block in the DM group. LB exhibits a bimodal pharmacokinetic profile with an initial plasma peak concentration at 1 h after administration and a second peak at 12–36 h [76]. We observed in our study that oscillations of the intensity of sensory block are less pronounced in the diabetic group that received LB. The clinical significance of this finding warrants further investigation. As is the case with conventional local anesthetics, the consistency and the prolonged duration of the peripheral nerve block in the diabetic mice with peripheral neuropathy treated with LB can be explained by the increased nerve sensitivity to local anesthetics, altered nerve conduction velocity, and the presence of microangiopathy that may delay local anesthetics absorption and uptake. Meanwhile, a few case reports [77,78] have described clinically important vasodilatory effects of LB in patients diagnosed with digital ischemia, a condition that may mimic tissue changes seen in diabetic peripheral neuropathy. The significance of this vascular effect in the context of diabetic peripheral neuropathy warrants further investigation.

### Limitations

Our study was carried out on a female mouse model, and mindful that sex differences in STZ sensitivity have been described in rodent models [79], another study on a male model is indicated. Additionally, while the results may be more reproducible, the STZ-induced diabetic mouse model does not correlate well with all aspects of DM type 1 or 2 in humans [80]. A high-fat-diet-induced diabetic mouse model might be more representative of the more prevalent DM type 2 in humans. We did not serially assess for the presence of neuropathy following STZ-treatment and thus could not determine the specific time of onset of peripheral neuropathy in the diabetic mice and if all mice developed signs of neuropathy at the same interval, and the implications of this to local anesthetic block duration. Considering the findings of subclinical neuropathy in patients with DM type 1 [81], it would be interesting to investigate the duration of local anesthetic block in diabetic mice without clinical evidence of neuropathy. Meanwhile, while the assessment tests for peripheral neuropathy in our study are considered valid [82], a more objective methodology would be the measurement of sensory conduction velocity and amplitude of sensory nerve action potential in the tail nerves [83]. While previous studies have measured the duration of analgesic effects of LB administered by different methods and in different mouse sites [55], data on the duration of sciatic nerve block after application of LB remains scarce. Of the four investigated duration times, three were within the interval set to determine the sensory block duration, while in the fourth for mice with DM and peripheral neuropathy, the duration of LB was above the already established interval. This could be a limitation of our study; however, as the purpose of the study was not only to determine the duration but to investigate the pattern of behavior and difference in duration, the applied statistical method allows us to compare the different durations and provide important conclusions.

## 5. Conclusions

Our results and analysis demonstrate that while LB provided a longer sensory block than BH in both control and diabetic mice, diabetic peripheral neuropathy significantly increased the duration, and intensity of nerve block with LB. Since the degree of peripheral neuropathy significantly affects the duration and intensity of sensory block, the type and concentration of local anesthetics should be considered very carefully on an individual basis. Given the increased nerve sensitivity in peripheral neuropathy, the standard concentration of 1.3% LB could be excessive; therefore, investigating different concentrations of local anesthetics would be of great value. In the presence of peripheral neuropathy, where acute postoperative pain can be intertwined with chronic pain, various modifications of nano-structured systems can widen the use of local anesthetics. Extended-release formulations with different encapsulated concentrations of local anesthetics for different nerve conditions might be a rational perspective for pharmaceutical research. Meanwhile, further studies are warranted to provide conclusive evidence on the efficacy and safety profile of the available extended-release local anesthetic formulations, including sensory and motor evaluation in different medical conditions [84]. We believe that our data could be useful to inform future studies and encourage investigators to design more robust studies on the effects of LB and other forms of prolonged-release local anesthetics in diabetic peripheral neuropathy.

## Figures and Tables

**Table 1:** Description of the sample characteristics in the control mice and mice induced with STZ for both treatments.

Variable	Total	Control			Induction with STZ		
		BH	LB	Total	BH	LB	Total
**Sensory design N (%)**	24 (100)	6 (50.0)	6 (50.0)	12 (100)	6 (50.0)	6 (50.0)	12 (100)
**Mean (SD) blood glucose before STZ (mmol/L)**	7.27 (0.8)	6.15 (0.9)	7.35 (0.5)	6.8 (1.0) Ⴕ	7.4 (1.4)	7.4 (1.0)	7.4 (1.2) Ⴕ
**Mean (SD) weight before STZ (g)**	23.8 (1.7)	23.8 (2.1)	24.4 (1.5)	24.1 (1.7) Ⴕ	23.3 (1.7)	23.7 (1.6)	23.5 (1.7) Ⴕ
Mean (SD) blood glucose after STZ * (mmol/L)	n.a.	8.0 (0.6)	7.3 (1.2)	7.6 (0.9) ‡	25.3 (5.9)	23.9 (5.2)	24.6 (5.4) ‡
**Mean (SD) weight after STZ (g)**	n.a.	29.0 (2.2)	28.3 (2.3)	28.5 (2.2) ‡	21.1 (1.6)	21.5 (3.3)	21.3 (2.4) ‡
**Plantar test (s)**	n.a.	6.1 (2.7)	6.7 (1.8)	6.4 (2.8) ‡	13.0 (3.3)	10.8 (3.4)	11.9 (3.4) ‡
**Tail flick test (s)**	n.a.	1.9 (0.4)	1.9 (0.4)	1.9 (0.4) †	2.0 (0.2)	2.2 (0.6)	2.2 (0.4) †
**Model sample N (%)**	24 (100)	6 (50)	6 (50)	12 (100)	3 (37.5)	5 (62.5)	8 (100)

Note: n.a. not applicable; * measurement before experiment; differences in the measured variables between the samples were analyzed using independent t-test; Ⴕ not significant; † significance at *p* < 0.1; ‡ significance at *p* <0.001.

**Table 2:** Differences between model predictions based on the duration pattern of different types of local anesthetics and different animal model conditions.

*Model*		*Obs.*	*ROC Area*	*Std. Err.*	*95% Conf. Interval*		*χ^2^*	*p-Value*
***LA***								
*** Model 1***	BH	20	0.56	0.137	0.29	0.83	5.3	0.022
*** Model 2***	LB	20	0.88	0.010	0.69	1.00		
***Condition***								
*** Model 1***	No condition	20	0.68	0.128	0.43	0.93	5.1	0.023
*** Model 2***	PN due to DM	20	0.92	0.068	0.78	1.00		

DM—diabetes mellitus, PN—peripheral neuropathy, LA—local anesthetic.

## Data Availability

The datasets used and analyzed during the present study are available from the corresponding author on reasonable request.

## Supplementary Materials

The following supporting information can be downloaded at: https://www.mdpi.com/article/10.3390/pharmaceutics14091824/s1, Table S1: Relationship between mean % MPE and time sequence (13.5 min) for control mice and mice with peripheral neuropathy due to DM treated with bupivacaine hydrochloride (BH) and liposomal bupivacaine (LB).

## List of Abbreviations

AMDCC	Animal Models of Diabetic Complications Consortium
BH	Bupivacaine hydrochloride
DM	Diabetes mellitus
LA	Local anesthetic
LB	Liposomal bupivacaine
MPE	Maximal possible effect
PN	Peripheral neuropathy
ROC	Receiver operating curves
SD	Standard deviation
STZ	Streptozotocin
