# Supplementary material for: The Influence of Diabetic Peripheral Neuropathy on the Duration of Sciatic Nerve Block with 1.3% Liposomal Bupivacaine and 0.25% Bupivacaine Hydrochloride in a Mouse Model
Source: Pharmaceutics. 2022 Aug 30;14(9):1824. doi: 10.3390/pharmaceutics14091824 (PMC9502724; doi:10.3390/pharmaceutics14091824)
Supplement: Supplementary file 1 [file pharmaceutics-14-01824-s001.zip › pharmaceutics-1851676-supplementary.pdf]

Supplementary Material

Supplementary Table S1. Relationship between mean % MPE and time sequence (13.5 min) for control mice and mice with peripheral neuropathy due to DM treated with bupivacaine hydrochloride (BH) and liposomal bupivacaine (LB).

		BH		LB	
Time point	min	Control<br>Mean MPE % (SD)	PN due to DM<br>Mean MPE % (SD)	Control<br>Mean MPE % (SD)	PN due to DM<br>Mean MPE % (SD)
1	13.5	83.9 (23.9)	42.5 (51.7)	90.2 (24.2)	100 (0)
2	27	42.4 (47.6)	89.2 (18.8)	93.4 (16.3)	64.4 (41.8)
3	40.5	18.8 (40)	0 (0)	42.5 (34.2)	67.3 (37)
4	54	39.9 (33.8)	41.9 (52)	66.4 (30)	59.2 (53.5)
5	67.5	3.5 (8.6)	12.5 (21.6)	31.8 (40.4)	100 (0)
6	81	2.3 (5.5)	0 (0)	17.8 (16.4)	40 (54.8)
7	94.5	17.2 (40.6)	11.7 (20.3)	25.6 (34.1)	60 (54.8)
8	108	12.7 (25.5)	0.7 (1.2)	5.6 (12.3)	61.9 (52.4)
9	121.5	2.2 (2.5)	5.1 (8.8)	3.6 (6.7)	38.8 (48.3)
10	135	15 (15.6)	34.6 (48.3)	6 (14.4)	80 (44.8)
11	148.5	2.1 (5.1)	33.4 (57.8)	17.2 (25.2)	42.5 (52.7)
12	162	18.5 (22.2)	0 (0)	12.8 (21.8)	70 (44.8)
13	175.5	28.7 (21.3)	1.4 (2.5)	26.1 (34.1)	51.3 (45.5)
14	189	35.9 (46)	0 (0)	29.1 (31.2)	74.3 (43.4)
15	202.5	19.4 (22.6)	68.7 (54.4)	67.3 (37.2)	100 (0)
16	216	12.9 (17.1)	33.4 (57.8)	28.4 (33.1)	74.2 (35.5)
17	229.5	0.9 (2.2)	0 (0)	6.5 (14)	60 (54.8)
18	243	6.7 (13.3)	19.1 (28.8)	15.2 (21.1)	60 (54.8)
19	256.5	11.9 (22.2)	21 (22)	18.4 (30.8)	40 (54.8)
20	270	4.9 (8)	42.3 (51.8)	17.7 (27.2)	50 (57.8)

DM– Diabetes mellitus, PN– peripheral neuropathy, MPE – Maximal possible effect, SD – Standard deviation
